# Simultaneous analysis of distinct Omics data sets with integration of biological knowledge: Multiple Factor Analysis approach

**DOI:** 10.1186/1471-2164-10-32

**Published:** 2009-01-20

**Authors:** Marie de Tayrac, Sébastien Lê, Marc Aubry, Jean Mosser, François Husson

**Affiliations:** 1 CNRS UMR 6061, Université de Rennes 1, IFR 140, Faculté de Médecine, CS 34317, 35043 Rennes, France; 2 Medical genomics Unit, Department of Biochemistry and molecular genetics, CHU Rennes, France; 3 CNRS UMR 6625, Laboratoire de mathématiques appliquées, Agrocampus Rennes, France; 4 Transcriptomic platform, Ouest-Genopole®, IFR 140, Rennes, France

## Abstract

**Background:**

Genomic analysis will greatly benefit from considering in a global way various sources of molecular data with the related biological knowledge. It is thus of great importance to provide useful integrative approaches dedicated to ease the interpretation of microarray data.

**Results:**

Here, we introduce a data-mining approach, Multiple Factor Analysis (MFA), to combine multiple data sets and to add formalized knowledge. MFA is used to jointly analyse the structure emerging from genomic and transcriptomic data sets. The common structures are underlined and graphical outputs are provided such that biological meaning becomes easily retrievable. Gene Ontology terms are used to build gene modules that are superimposed on the experimentally interpreted plots. Functional interpretations are then supported by a step-by-step sequence of graphical representations.

**Conclusion:**

When applied to genomic and transcriptomic data and associated Gene Ontology annotations, our method prioritize the biological processes linked to the experimental settings. Furthermore, it reduces the time and effort to analyze large amounts of 'Omics' data.

## Background

Genome-wide analyses provide an unprecedented amount of data leading to new interpretation challenges. Classical microarrays can monitor the expression of potentially all genes within a cell or a tissue sample. More recently, new applications have been developed. They include chromatin-immunoprecipitation-chip (ChIP-on-Chip), analysis of alternative splicing (Exon array), characterization of the methylome, polymorphism genotyping (SNP array), copy-number measurements (CGH array) and genome resequencing (for review [1,2]). A great interest in the statistical analysis of these 'Omics' data has emerged and many methodologies have been developed. However, if the inferential statistics analyses are now guided by consensual methods [3,4], the descriptive analysis is often succinct if not neglected. Two reasons can be advanced: (i) the great volume of information makes difficult the interpretation of the results, and (ii) heterogeneous data and multiple sources of information are difficult to integrate in a global analysis. Methods that overcome these difficulties are necessary as the understanding of a biological phenomenon would greatly benefit from considering simultaneously several types of 'Omics' data and particularly with biological knowledge. This could be done in a multidimensional exploratory approach.

In a multidimensional exploratory approach, a microarray data set is usually analyzed by multivariate analysis (MVA) among which Principal Components Analysis (PCA) is the most used. PCA is well adapted to the framework of 'Omics' data as it can handle data sets with much more variables (genes) than samples (arrays). To analyze simultaneously several data sets, the proper way is to use MVA's dedicated to the analysis of multi-way data tables; the method of reference being the generalized canonical analysis (GCA) [5]. In the field of microarray, GCA is however limited by the problem of multi-colinearity. To bypass this limitation, only two alternatives have still been proposed: the generalized co-inertia analysis (CIA) [6-8] and the recently applied regularized canonical correlation analysis (RCCA) [9,10].

The need for integrating external information in MVA to ease the interpretation of microarray data have also been pointed out. As proposed by Busold *et al*. [11], Fagan *et al*. [12] superimposed Gene Ontology (GO) terms as supplementary elements onto CIA projections. In this study, GO terms are formalized as boolean vectors that are projected onto CIA plots after matrix transformations. Although CIA approach provide good results in combining molecular data sets, the way GO terms are added is not straightforward and appears incomplete. Indeed, this method codes the links between genes and GO terms and do not take into account the microarray values or molecular data of the genes related to each GO term. Other computational methods, such as gene set enrichment analysis (GSEA) [13], have shown the importance of focusing on groups of genes as opposed to individual genes for incorporating biological information and gene sets into microarray data analysis. Following this philosophy, a proper integration of biological information in MVA will gain in accuracy by grouping genes into knowledge-related modules, and thus by considering a 'modular approach' [14,15]. Such an approach studies as a whole the behavior and structure of a biological process in addition to analyzing its components (genes and/or gene products) individually.

In this article, we suggest to use Multiple Factor Analysis (MFA) in the sense of Escofier-Pagès [16,17] to integrate bio-molecular data sets as well as informations on the genes structured in modules. MFA is dedicated to the simultaneous exploration of multi-way data sets where the same individuals are described by several groups of variables. MFA is commonly applied to sensory and ecology data and it has already been applied to the analysis of metabonomic data [18]. MFA can be related to GCA and CIA since it could be considered as a particular generalized canonical analysis were the inertia criteria replaces the correlation criteria. These methods display a low-dimensional projection of the data highlighting the main sources of variability. Results should therefore be interpreted with caution as sources of variability are not always due to specific biological factors of interest. It is also important to note that at the sample level, the structures provided either by MFA or CIA are highly similar [6]. The assets of MFA appear when integrating both numerical and categorical groups of variables, and when supplementary groups of data need to be added in the analysis. Here, we present our approach by introducing the basis of MFA and we state how MFA is particularly well adapted to integrate formalized biological knowledge. We illustrate our method with a glioma study [19,20] performed with both CGH array and expression microarray on the same tumor samples. Results shows that both DNA copy number alteration and transcriptome data sets induce a good separation of the gliomas according to the WHO classification. The superimposition of the gene modules built since GO annotation identify regulatory mechanisms implicated in gliomagenesis. We also show that our approach can handle a single data set with associated GO annotations and therefore be used as an exploratory tool in the case of classical single 'Omics' study. Finally we present another illustration focused on a nutrition study in mice and integrating microarray and lipidomic data.

## Results and discussion

**Figure 1:**
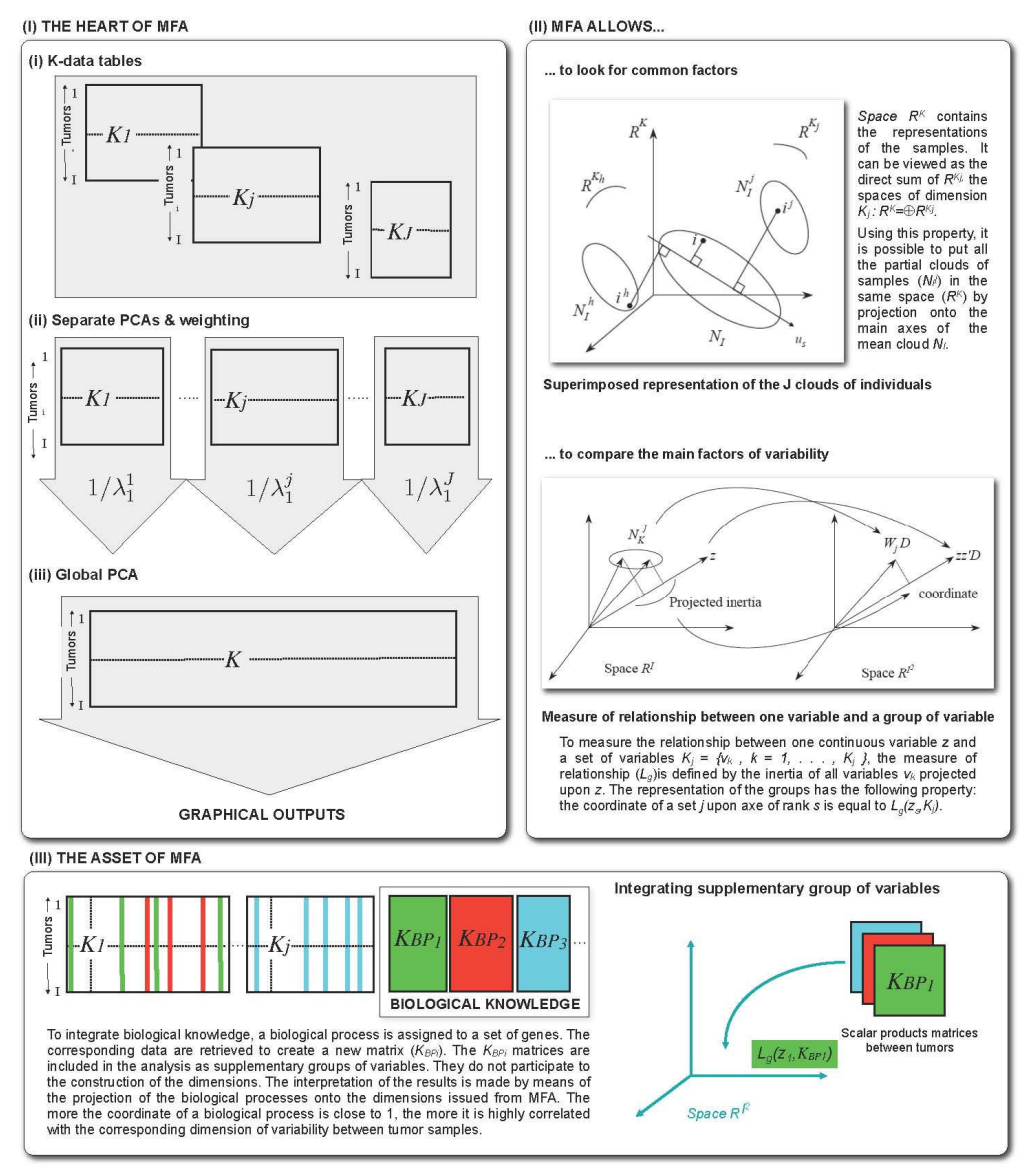
**Schematic of our MFA based approach to combine 'Omics' data and to integrate biological Knowledge**. (I) The heart of MFA is a PCA in which weights are assigned to the variables: (i) When several sets of variables describe a same set of individuals (tumors), it is possible to consider the merged data set: *K *= [*K*_1_, *K*_2_,..., *K*_*J*_], where each *K*_*j *_corresponds to an 'Omics' data table. (ii) Separate analysis are performed by principal components analysis (PCA) on each group *j *of variables. Each variable belonging to a group *j *is weighted by 1/$\lambda_{1}^{i}$, where $\lambda_{1}^{i}$ denotes the first eigenvalue of the matrix of variance-covariance associated with each data table *K*_*j*_. (iii) A global analysis is performed. The corresponding graphical displays (Individual Factor Map and Variables Representation) are read as for PCA. (II) MFA allows to look for common factors by providing a representation of each matrix of variables (Groups Representation). It provides the visualization of specific and common structure emerging from the *K*_*j*_. MFA allows to compare the main factors of variability by linking both groups and variables representations. As the coordinates of set *j *upon axis of rank *s *is equal to *L*_*g*_(*z*_*s*_, *K*_*j*_): set coordinates are always comprised between 0 and 1; and a small distance between two set along axis *s *means that they include the structure expressed by factor s each one with the same intensity. (III) The asset of MFA to add supplementary groups of variables is used to integrate biological knowledge. The BP modules are formalized as $K_{BP_{i}}$ matrices containing the restriction of the whole data set to the genes associated with the *i*^*th *^BP. The projection of the $K_{BP_{i}}$ is made by means of its scalar product matrix between individuals. This matrix denoted *W*_*i *_is a a (*I *× *I*) matrix (*W*_*i *_= $K_{BP_{i}}{K^{\prime }}_{BP_{i}}$) and can be considered as an element of the space ${\mathbb{R}}^{I^{2}}$. This element is thus projected on the dimensions of ${\mathbb{R}}^{I^{2}}$ issued from MFA. This representation of the groups is made available by means of a graphical display of the $K_{BP_{i}}$ as points in a scatter plot. It has to be read as follow: the coordinate of a given group is all the more close to 1 than the variables of this group are highly correlated with the dimension issued from the MFA (either positively or negatively). Hence, two groups are all the more close than the structures they induce on the observations are close.

MFA is used to analyze several groups of 'Omics' variables (numerical and/or categorical) defined on the same samples. The core of MFA is a PCA applied to the whole set of variables in which each group of variables is weighted, rendering possible the analysis of different points of view by taking them equally into account. To illustrate the main features of our approach a schematic is provided in Figure 1. Each time, MFA is applied to published-data selections (see the section *Data and notations *in the *Materials and Methods*). Resulting graphical outputs for the first two principal components (PC1 and PC2) are used. We describe the results and discuss the following points:

(i) the combination of paired CGH array and microarray data of glial tumors;

(ii) the assemblage of genes into modules based on Gene Ontology terms and their superimposition on the principal components issued of point (i), supporting interpretation of the study;

(iii) the application of our approach in the case of a single 'Omics' study (transcriptome of gliomas);

(iv) an other illustration of our method with a different experimental setting using a nutrition study including microarrays and fatty acids gas chromatography data.

The analyses have been performed with R and the package FactoMineR [21].

### Multi-way glioma data set

#### Joining 'Omics' distinct points of view (genome and transcriptome)

**Figure 2:**
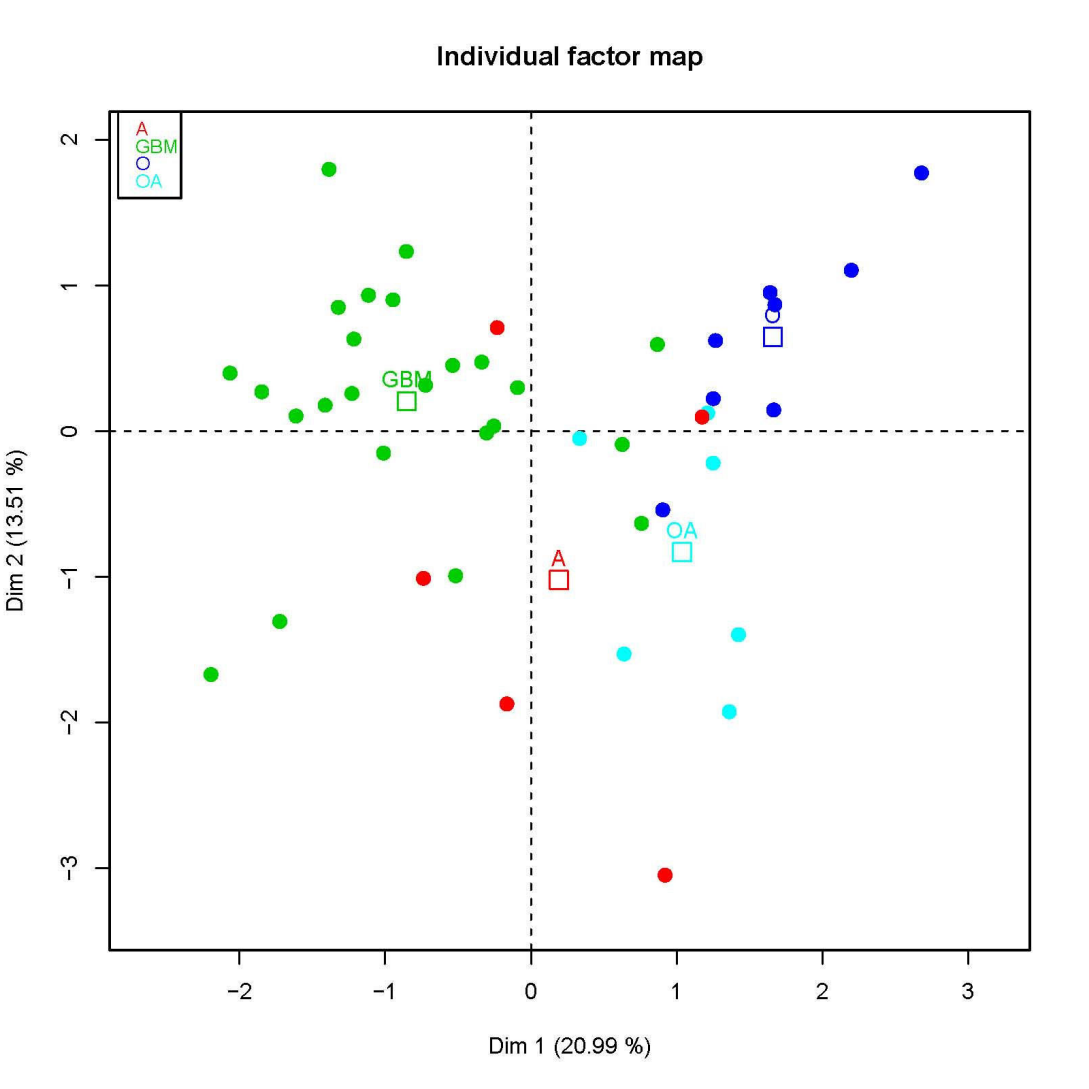
**Multi-way glioma data set: MFA consensus between CGH and expression highlights a partition of gliomas into WHO classification**. Individuals (tumors) are presented as points on the scatter plot created with the first two main dimensions of MFA. Each individual is colored following the glioma subtype (WHO classification); mean individual are also displayed. Projection of the tumors onto PC1 underlines a partition into glioblastomas (GBM) and lower grade gliomas (oligodendrogliomas, astrocytomas, oligoastrocytomas). PC2 mainly stresses differences between astrocytomas (A) and oligodendrogliomas (O). As PC1 and PC2 represents the first two main factors of MFA they could be interpretated: PC1 summarizes *characteristics of glioblastoma i.e*. transcriptional differences existing between glioblastomas and lower grade gliomas; PC2 summarizes *characteristics of oligodendrogliomas *as it stresses the differences between glial tumors coming from astrocytic cells from those arising from oligodendroglial ones.

**Figure 3:**
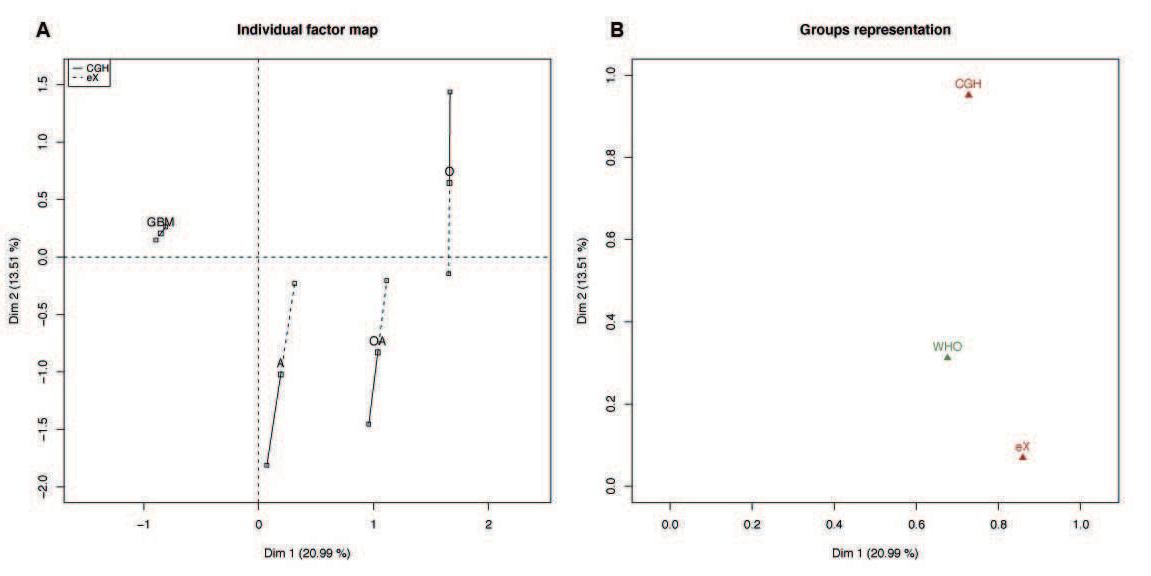
**Multi-way glioma data set: Characteristics of glioblastoma are linked to CGH and expression data whereas characteristics of oligodendrogliomas are mostly related to CGH data**. The partial representation of the mean individuals (CGH and eX) for each WHO tumor type (A) and the group representation (B) are displayed. (A) The balanced representation of each category is located in the exact barycenter of the points summarizing partial points of view (CGH; linked by plain line and eX; linked by dot line). The projection of the partial representations for each category (CGH and eX for oligodendrogliomas, astrocytomas, oligoastrocytomas and glioblastomas) onto PC1 are very close; the partition of the tumors into WHO classification is thus shared by the genome and the transcriptome. On PC2, all the mean individuals from the partial expression representation (eX) are located around the origin. It is not the case for the genomic one (CGH). PC2 is therefore specific to the genomic point of view and is not shared by the expressional one. This is confirmed by analyzing the group representation (B): projection of the CGH and eX groups are closed along PC1 but only the one of CGH have a value close to 1 on PC2.

MFA is applied to the paired CGH array and microarray glioma data of Bredel *et al*. [19,20]. The resulting sample plots (33.7% of the total variability) are presented Figure 2 and Figure 3. The mean representation of the samples according to both CGH and gene expression data sets is presented Figure 2. Mean samples are represented by points colored following WHO classification of the tumors. Figure 3A shows the partial representation associated to each type of tumors (WHO classification: O, oligodendrogliomas; A, astrocytomas; OA, mixed oligo-astrocytomas and GBM, glioblastomas). This representation is obtained from the consensus between the CGH and expression (eX) points of view (i.e. genome and transcriptome variations). Each type of tumors is represented by three points: the consensus between the two points of view and a point for each point of view. Both scatter plots show a well-defined partition of the samples into WHO classification. This is particularly true along PC1 that underlines a partition of the samples into glioblastomas (GBM) and lower grade gliomas (O, A, OA). Partial representation (Figure 3A) and groups representation (Figure 3B) show that this partition exists (i) on PC1 at the genome and at the transcriptome levels and (ii) only at the genome level on PC2. Indeed, the projections on PC1 of the partial points for each category of tumors (CGH and eX for O, A, OA and GBM) are each time very close, meaning that CGH and eX define similar structures upon tumors on PC1. In a same manner, the projections of groups CGH and eX on PC1 have coordinates close to 1. On PC2, all the mean individuals from the partial expression representation (eX) are located around the origin, which is not the case for the genomic one (CGH); meaning that PC2 is specific to the genomic point of view. In the same manner, only projection of group CGH on PC2 has coordinates close to 1 (Figure 3B). Regarding CGH data, PC2 provides a partition of the histological subtypes and particularly stresses differences between oligodendrogliomas (O) and astrocytomas (A). The one-variable group WHO summarizing the tumor classification is projected as an illustrative group (Figure 3B). Since its coordinate on PC1 is rather high, the structure induced by this group is linked to PC1: the types of tumors are well separated along this dimension. Its coordinate on PC2 is also relatively important, showing that the types of tumors are also separated on PC2. Following the examination of these graphical outputs, PC1 is linked to *glioblastoma characteristics *and PC2 corresponds to *oligodendroglioma characteristics *as it stresses the differences between these tumors and the other gliomas.

**Figure 4:**
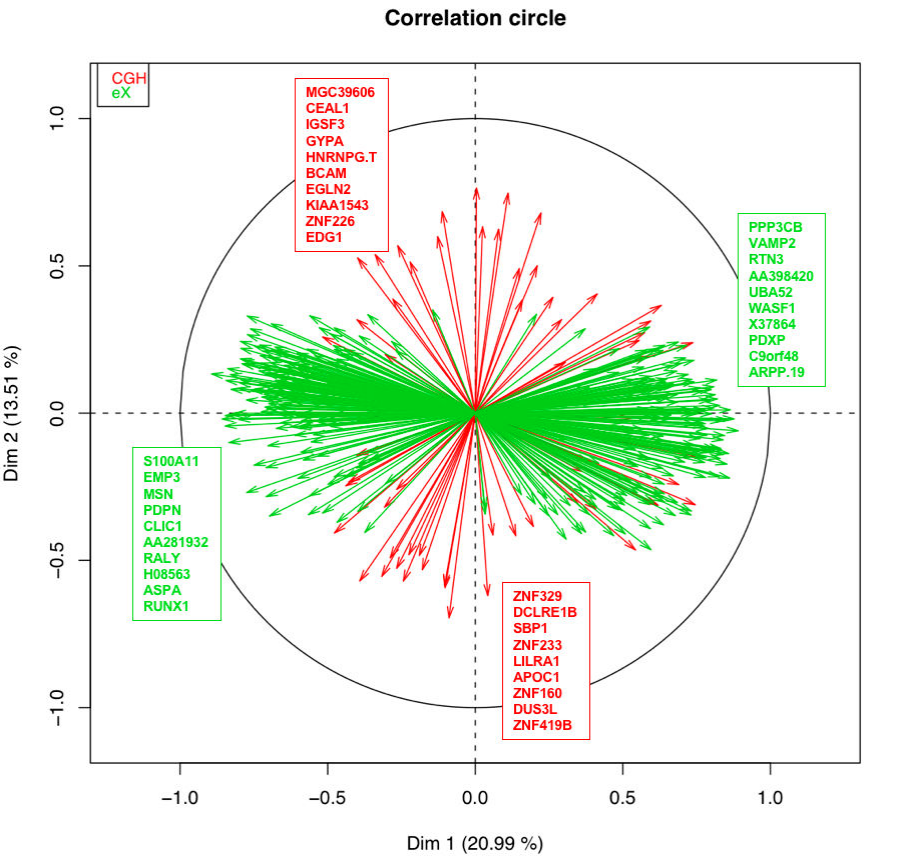
**Multi-way glioma data set: Characteristics of oligodendrogliomas are linked to modifications of the genomic status of genes located on 1p and 19q positions**. The Variables Representation is displayed and the genes that are the most correlated to each principal components (ten positively and ten negatively) are named. Each genes of the two groups (CGH, red and eX, green) are projected on the factor map and represented as a vector. This scatter plot is read as in PCA. The more a vector has a magnitude close to 1, the best the projection is. The vector points in the direction of the high values, e.g. in our case, a gene from eX corresponding to a vector pointing in the right side is over expressed in oligodendroglioma compared to glioblastoma. The examination of the genes linked to PC2 underlines modifications of the genomic status of genes located on 1p and 19q positions (see Table 1).

Beyond the partition of the tumors, the challenge lies in interpreting the results to gain insights into biological mechanisms. The classical way is to identify the genes most correlated with each principal component, see Variables Representation (Figure 4). This scatter plot representing the genes is read as in PCA. Briefly, the genes are projected on the factor map and represented as vectors. The more a vector has a magnitude close to 1, the best the projection is. The vector points in the direction of the high values, e.g. in our case, a gene with a corresponding vector pointing in the right side takes higest values in oligodendrogliomas compared to glioblastomas. We thus retrieved the genes most correlated with PC1 and PC2. Once the identifiers recovered, they can be annotated manually by gathering functional information from a large panel of databases and annotation tools. For example, the manual examination of the genes linked to PC2 underlines genomic status modifications of genes located on 1p and 19q positions (Table 1). Allelic alterations of chromosomes 1 (short arm) and 19 (long arm) are frequently reported as important events in gliomas [22] and especially in oligodendrogliomas [23]. Indeed, it is reported that these chromosomal aberrations patterns vary according to the categories of glial neoplasms and could be marks of malignant progression [24]. This process is however laborious and highly time consuming. Moreover, the interpretation of such emphasized structures remains difficult when only associated gene IDs are accessible or when lots of genes have to be taken into account. For that reason, providing gene annotations in a corresponding plot is necessary to obtain a concise way of understanding these results.

**Table 1:** Multi-way data set: Genes strongly correlating with characteristics of oligodendrogliomas.

Gene Symbol	Chromosome
ZNF233	19q13.31
APOC1	19q13.2
ZNF329	19q13.43
DCLRE1B	1p11.1
LILRA1	19q13.4
EDG1	1p21
ZNF226	19q13.2
GPSM2	1p13.3
ZNF549	19q13.43
KIAA1543	19p13.3-p13.2
IGSF3	1p13
BCAM	19q12-q13
ISOC2	19q13.42
EGLN2	19q13
CEAL1	19q13.31

The first 15 genes most strongly correlating with PC2 are shown with identifiers and corresponding chromosome locations. PC2 stresses differences between oligodendrogliomas (O) and other gliomas and is linked to the modifications of the genomic status of genes located on 1p and 19q. This highlights that oligodendrogliomas could be associated with a specific allelic alterations of chromosomes 1 (short arm) and 19 (long arm).

#### Integrating biological knowledge: superimposition of GO gene modules

As we pointed out, the interpretation of the structures emerging from MFA constitute a difficult and time-consuming step. Our approach aims at easing this task. Firstly, the biological knowledge is formalized. Here, Gene Ontology (GO) biological process (BP) terms are used to assemble gene modules. Secondly, the gene modules are superimposed on the same principal components and aid interpretation of the study. This is allowed by the capacity of MFA to integrate supplementary groups of data. The results has to be read as follow: the coordinate of a given group is all the more close to 1 than the variables of this group are highly correlated with the dimension issued from the MFA. Hence, two groups are all the more close than the structures they induce on the observations are close. Thus, the coordinate of one gene module provides a direct measure of the link between its constituting genes and the corresponding principal component (*glioblastoma characteristics *for PC1 and *oligodendroglioma characteristics *for PC2).

**Figure 5:**
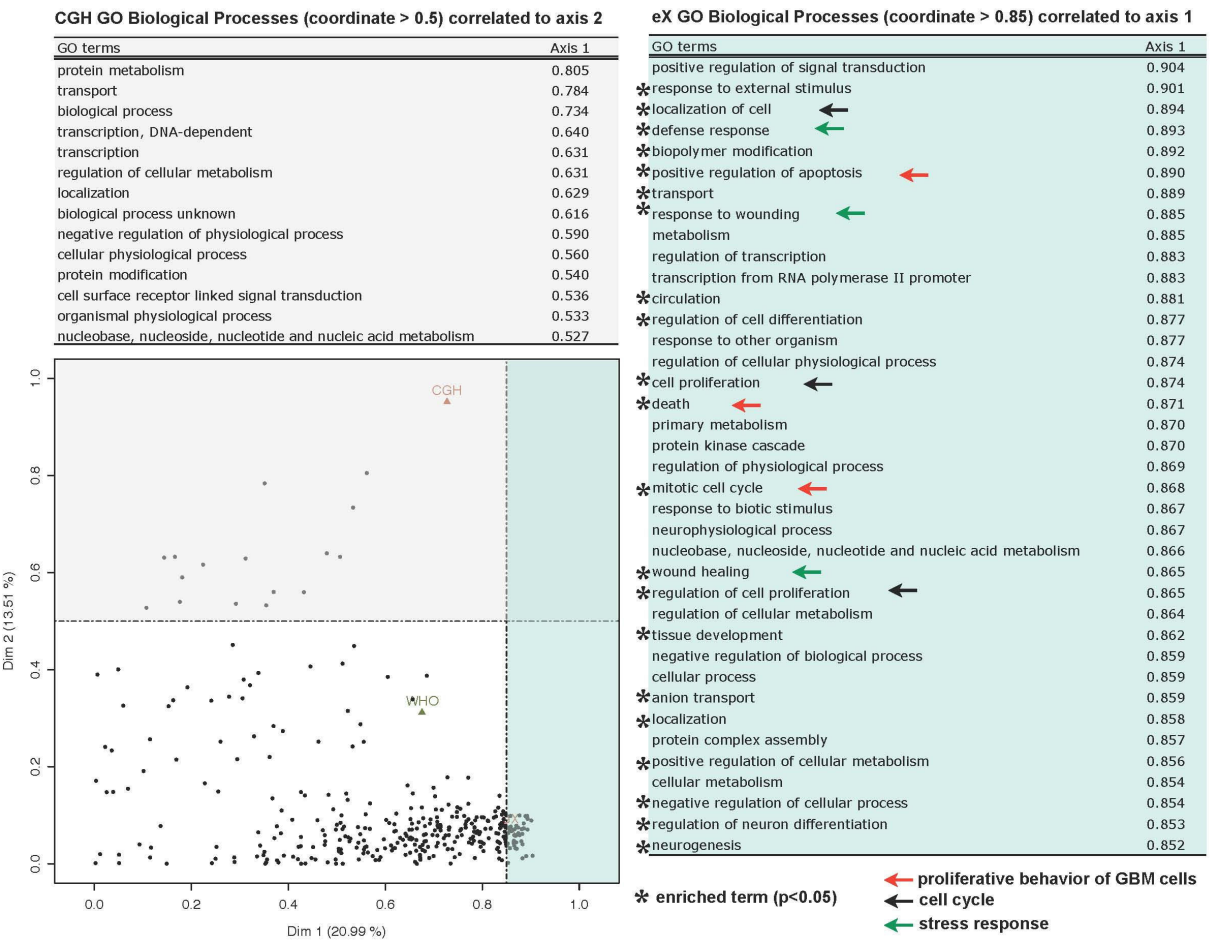
**Multi-way glioma data set: Superimposing biological knowledge underline hallmark of glioblastomas and potential markers for glioma diagnostic**. Each group of variables is projected on the factor map created by MFA: active groups (points) and gene modules (triangles) are plotted. Only GO identifiers representing modules highly linked to the dimensions 1 and 2 are displayed. To facilitate the interpretation of the plot, only GO terms representing modules highly linked to PC1 (blue box, *coordinate *≥ 0.85) and 2 (grey box, *coordinate *≥ 0.5) are displayed. Enriched terms (*p*-value < 0.05) are marked with a star. The qualitative group WHO classification is also shown.

Figure 5 provides a typology of the modules and highlights shared dimensions between GO BP terms and tumor groups. To facilitate the interpretation of the plot, the GO terms with highest coordinates (> 0.85 for PC1 and > 0.5 for PC2) are listed on separated boxes (blue for PC1 and grey for PC2). PC1 or *glioblastoma characteristics *is thus interpreted thanks to its BP-associated list. As example, three main BP categories are particularly represented. The first one supports the proliferative behavior of GBM cells (red arrows) with GO terms like 'localization of cell' (GO:0051674), 'cell proliferation' (GO: 0008283), and 'regulation of cell proliferation' (GO:0008284). The second one is related to the cell cycle (black arrows) with 'positive regulation of apoptosis' (GO:0043065), 'death'(GO:0016265), and 'mitotic cell cycle' (GO:0000278). Eventually, a third one indicates a link with the response to a stimulus, particularly stress and defense (green arrows): 'defense response' (GO:0006952), 'response to wounding' (GO:0009611), and 'wound healing' (GO:0042060). These annotations underline the hallmark of glioblastomas: a rapid progression with cell cycle dysfunctions, important angiogenesis and highly proliferative and invasive tumor cells. The factor delineating oligendroglial tumors from the other gliomas (PC2) is mainly associated with modules related to transport and to transcription processes. Among these biological processes, 'protein metabolism' (GO:0019538), 'transport' (GO:0006810), and 'transcription DNA-dependent' (GO:0006350) annotate 18 genes of those located on 1p or 19q. The homogeneity and coherence of these modules associated with targeted damages of the genome structure appear as potential cumulative events. They appear to be distinctive features of oligendroglioma and could therefore constitute reliable markers for glioma diagnostic.

### Single glioma data set

We used the same approach to analyze the transcriptome data from Freije *et al*.. To achieve this, we applied MFA to the duplicated data set. Performing such task is strictly equivalent to applying a PCA on the initial data set. The results provided correspond to those obtained with a PCA and it becomes possible to manage additional informative groups of variables. To analyze the results, we follow a step-by-step interpretation of the principal components: based firstly on the typology of the tumors, secondly on the gene expression signatures and then, on the associated biological knowledge.

**Figure 6:**
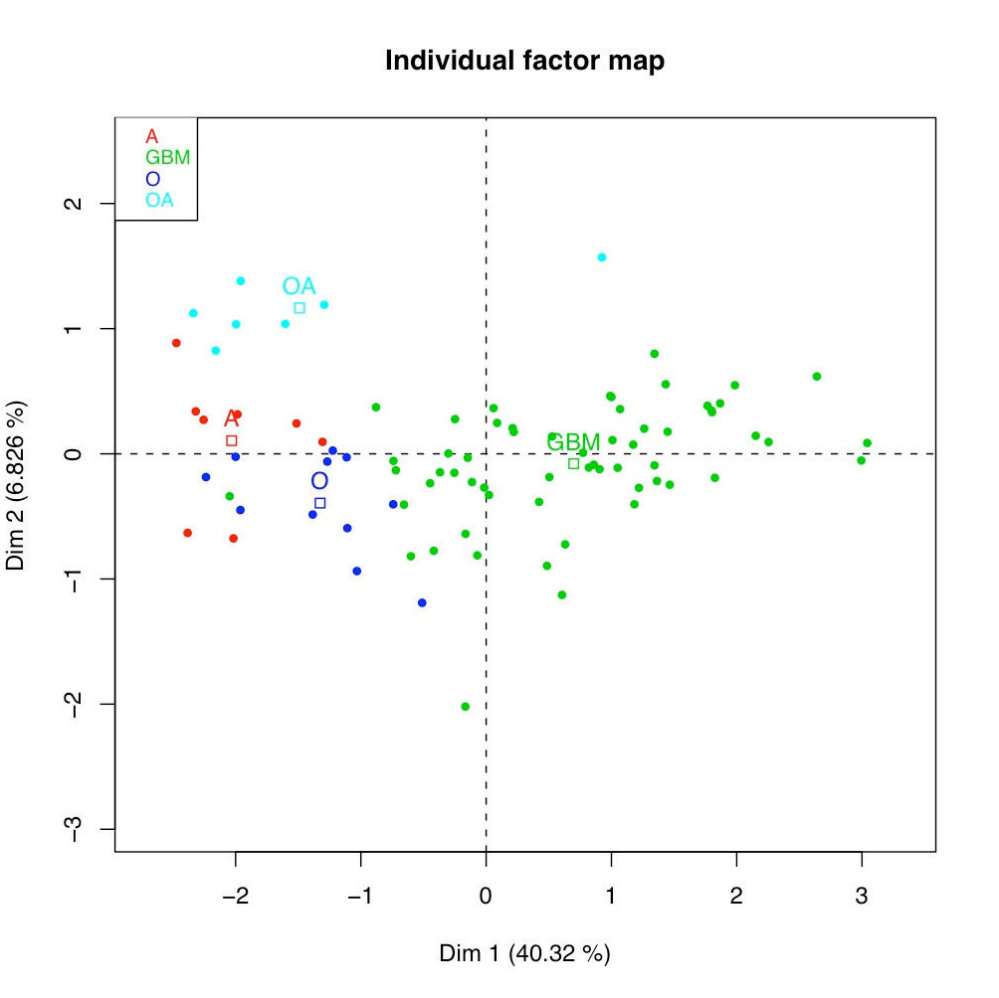
**Single glioma data set: MFA highlights a good separation between glioblastomas and lower grade gliomas**. Tumors are presented as points on the scatter plot created with the first two main dimensions of MFA. Each sample (dot) is colored following the glioma subtype (WHO classification); mean individual are also displayed (squares). Projection of the tumors onto PC1 underlines a separation between glioblastomas (GBM) and lower grade gliomas (oligodendrogliomas, astrocytomas, oligoastrocytomas). PC2 differentiates oligo-astrocytomas (OA) from the other subtypes (oligodendrogliomas, astrocytomas, GBM). PC1 is linked to *characteristics of glioblastoma *stating transcriptional differences between grade IV and lower grade gliomas. PC2 is related to *characteristics of oligodendrogliomas *highlighting OA particular signatures.

We focus on the first two principal components that explain 47.1% of the total variability carried by the 615 genes. The corresponding individuals factor map is provided in Figure 6. Mean observations are added for each glioma subtype to help with the interpretation of the plot. This map shows a relatively well-defined partition of tumors into WHO classification. It also shows that the position of the samples belonging to a glioma subtype varies from one to another. This variability could be assigned to the well known cellular heterogeneity of gliomas and particularly of glioblastomas. It could also be the result of the WHO classification that is somehow controversial: this standard classification is said to suffer from a lack of reproducibility among pathologists [25]. The projections on PC1 of the mean observations underline that the maximum of variability captured in the analysis separates glioblastomas (GBM) from lower grade gliomas (O, A, OA). PC2 differentiates oligo-astrocytomas (OA) from the other subtypes (O, A, GBM). As a result, PC1 is linked to *glioblastoma characteristics *stating transcriptional differences between grade IV and lower grade gliomas and PC2 is related to *oligoastrocytoma characteristics*, highlighting OA particular signature.

To assign a meaning, based on gene expression, to the *glioblastoma characteristics*, we retrieved the ten genes most strongly correlated with the first axis (Table 2). These genes were manually annotated by gathering some information from PubMed and were analyzed using the Ingenuity Pathway Analysis (IPA, Mountain View, CA). Among these ten genes, four are co-cited with 'glioma' on PubMed and five with 'cancer'. And the IPA analyses gives links between ANXA2, CLIC1, HEXB, MAPT and S100A11 in a network related to 'Cellular Movement, Cell-To-Cell Signaling and Interaction, Nervous System Development and Function'. These findings are in adequacy with the invasive nature of glioblastoma compare to lower grade glioma *i.e*. with *glioblastoma characteristics*. However, this interpretation step is partial as it was limited to only few genes; it thus will gain by integrating a global view of the associated knowledge.

**Table 2:** Single data set: Genes strongly correlating with gliobastoma.

Gene Symbol	Gene Name	PC1
S100A11	S100 calcium binding protein A11	0.895
ANXA2	annexin A2	0.876
CLIC1	chloride intracellular channel 1	0.871
HEXB	hexosaminidase B (beta polypeptide)	0.868
MRC2	mannose receptor, C type 2	0.867
FRY	furry homolog (Drosophila)	-0.834
PHYHIPL	phytanoyl-CoA 2-hydroxylase interacting protein-like	-0.848
CLASP2	cytoplasmic linker associated protein 2	0.851
GLUD1	glutamate dehydrogenase 1	-0.855
MAPT	microtubule-associated protein tau	-0.874

The first 10 genes most strongly correlating with PC1 are shown with identifiers and corresponding coordinate. Among these ten genes, four are co-cited with 'glioma' on PubMed and five with 'cancer'. And the Ingenuity Pathway Analysis gives links between ANXA2, CLIC1, HEXB, MAPT and S100A11 in a network related to 'Cellular Movement, Cell-To-Cell Signaling and Interaction, Nervous System Development and Function'.

**Figure 7:**
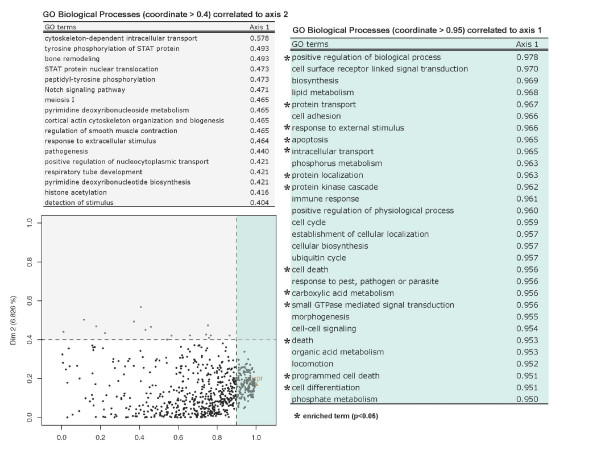
**Single glioma data set: Superimposing biological knowledge underline genes potentially involved in gliobastoma**. Each group of variables is projected onto the factor map created by MFA: active group or gene expression data set (triangle) and supplementary groups or gene modules (points) are plotted. To facilitate the interpretation of the plot, only GO terms representing modules highly linked to the PC1 (blue box, *coordinate *≥ 0.95, *number of annotated genes *<*50*) and PC2 (grey box, *coordinate *≥ 0.4) are displayed. Enriched terms (*p*-value < 0.05) are marked with a star.

We superimpose the associated biological knowledge as GO modules on the previously analyzed plots. The corresponding map (Figure 7) provides a typology of the functional modules and highlights shared dimensions between GO BP terms and tumor groups. As seen before, the coordinates of the annotation groups onto each principal component provide a direct measure of the links between modules and the corresponding factors (i.e., *glioblastoma characteristics *for PC1 and *oligoastrocytoma characteristics *for PC2). We focus on the gene modules linked to the glioblastomas and retrieved 49 direct GO terms strongly associated with grading (PC1: *coordinate *≥ 0.9). Among them, 27 GO terms were picked as they can be grouped into three main biological process categories (Table 3). The first main category is related to cell death and cell cycle regulation and the second one stresses that glioblastoma have a particular proliferative behavior. The third one indicates a link with developmental functions, more precisely in brain. These groups of annotations underline the well known characteristics of glioblastomas among other gliomas: a rapid cellular proliferation sustained by cell cycle dysfunctions and invasion of the parenchyma by isolated tumor cells. Moreover, our approach emphasizes the existence of a relation from neurogenesis-related genes to glioma grades of malignity. This supports the evidence that glioblastomas contain and may arise from neural stem cells or from differentiated cell types that display multipotential stem cell-like properties.

**Table 3:** Single data set: Gene Ontology terms highly associated with gliobastoma

Enrich. GO Identifier	Go Term	PC1
*Cell cycle/Death*		
* GO:0016265	death	0.953
GO:0007049	cell cycle	0.958
* GO:0008219	cell death	0.956
* GO:0012501	programmed cell death	0.951
GO:0051726	regulation of cell cycle	0.941
* GO:0006915	apoptosis	0.965
GO:0000074	regulation of progression through cell cycle	0.921
* GO:0042981	regulation of apoptosis	0.923
GO:0045786	negative regulation of progression through cell cycle	0.904
GO:0007050	cell cycle arrest	0.912
		
*Proliferative behavior*		
* GO:0051179	localization	0.986
* GO:0007154	cell communication	0.985
GO:0007155	cell adhesion	0.966
GO:0040011	locomotion	0.952
* GO:0051234	establishment of localization	0.985
GO:0007267	cell-cell signaling	0.954
GO:0007626	locomotory behavior	0.919
* GO:0008283	cell proliferation	0.917
GO:0051641	cellular localization	0.918
* GO:0042127	regulation of cell proliferation	0.916
* GO:0006928	cell motility	0.916
		
*Development*		
GO:0007275	multicellular organismal development	0.984
GO:0009653	anatomical structure morphogenesis	0.955
* GO:0030154	cell differentiation	0.951
* GO:0048513	organ development	0.942
GO:0007399	nervous system development	0.943
* GO:0000902	cellular morphogenesis	0.926

The annotation are grouped by meaningful categories (*Cell Cycle/Death; Invasive behavior *and *Development*) and are ordered by GO depth. PC1 coordinates for each GO annotation is provided in the third column. These groups of annotations underline the well known characteristics of glioblastomas among other gliomas: a rapid progression with parenchyma invasion by isolated tumor cells sustained by cell cycle dysfunctions. It also emphasizes the existence of a relation from neurogenesis-related genes to gliobastoma. Enriched terms (*p*-value < 0.05) are marked with a star.

The factor delineating the oligo-astrocytic tumors from the other ones (PC2) is particularly associated with modules related to the cytoskeleton. It is thus possible to highlight 'cytoskeleton-dependent intracellular transport' (GO:0030705), 'cortical actin cytoskeleton organization and biogenesis' (GO:0030866) and 'positive regulation of nucleocytoplasmic transport' (GO:0046824). These biological processes stress that the main dissimilarity existing between OA and other gliomas may emerge from a transcriptional modification of genes linked to actin cytoskeleton which is one of the possible determinants of human astrocytoma migration [26]. Seven genes are annotated with these GO BP terms: DYNC1H1, EPB41L2, KIF3A, KIF5B, MYL6, MYO9B, TRIP6. Among them, some are reported to be involved in cancer cell migration. Specifically, cytoskeleton rearrangements have been shown to be induced by a down-regulation of TRIP6 in carcinoma cell lines [27]. It was also suggested that MYL6 could have an effect on the migration of breast cancer cells [28]. Thus, the gene EPB41L2 codes a protein belonging to the protein 4.1 family which is proposed to have roles in human cancer [29]. And an increased expression of KIF3A may also be associated with Autocrine motility factor-induced signaling for cell motility and metastasis [30]. To our knowledge, none of these genes have been so far shown to be involved in glioma invasiveness.

### Multi-way nutrimouse data set

In this section we describe another scenario with controlled experimental design where biological units (mice) are cross-classified according to two factors: Genotype (wild-type (WT) versus PPAR*α *deficient mice (PPAR)) and Diet (5 diets with different fatty acid (FA) compositions). The measurements come from two different sources of information: transcriptome variables and hepatic FA measurements. With this example, we wish to illustrate the relevance of MFA-based interpretations in accordance with the conclusions drawn by specialists in [31].

**Figure 8:**
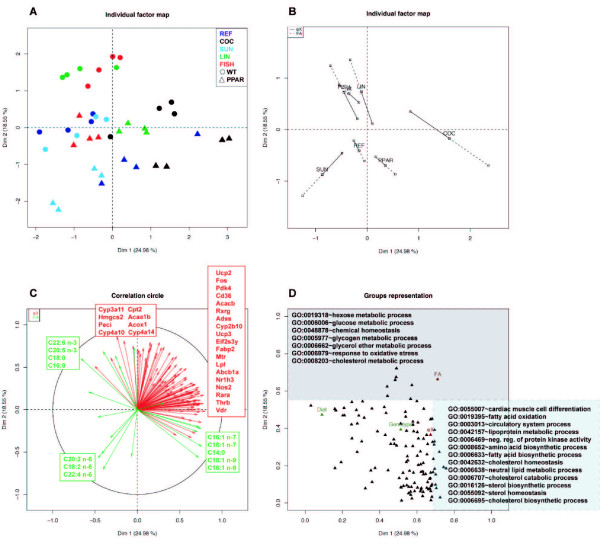
**Multi-way nutrimouse data set: MFA approach provides findings in accordance with those found by specialists**. A and B. Individual factor maps show partition of the mice according to Diet and Genotype. Mice fed the COC diet (black circles and triangles) are separated from the other mice on PC1. PPAR*α *deficient mice (triangles, PPAR) are separated from wild-type mice (circles, WT) on PC2. C. Variables representation. The genes and FAs most correlated to each principal components are named. Mice from both genotype displayed specific accumulations of FA families. Mice fed the SUN diet had enriched proportion of n-6 FAs, those fed the LIN and FISH diets are particularly related to n-3 FAs. Mice fed the COC diet preferentially accumulate mono-unsaturated FAs in their livers. Genes most correlated with PC2 are PPAR*α *target genes (Peci, Cyp4a10, Cyp4a14, Acox1, Acaa1b, Hmgcs2, Cpt2) or genes involved in hepatic detoxification (Cyp3a11). D. Enriched GO terms (*p*-value < 0.05) are projected on the Groups scatter-plot, the most correlated to each PC are named. Annotations related to PC1 and PC2 are respectively linked to FA and glucose metabolism. The GO term 'cardiac muscle cell differentiation' (GO:0055007) is enriched by class II nuclear receptors (retinoid × receptors and retinoic acid receptors) which are also key regulators of lipid metabolism.

Figure 8 shows the graphical results of our approach applied to the nutrimouse data set. The individual factor maps (Figure 8A) separates the mice according to Diet (PC1) and Genotype (PC2). Mice fed the COC diet are separated from the other mice on PC1. PPAR*α *deficient mice are separated from wild-type mice on PC2. This PC also highlights LIN and FISH diet. Partial (Figure 8B) and groups representation (Figure 8D) show that this partition of mice on PC2 is particularly due to FA data. Mice from both genotype displayed specific accumulations of FA families that were present in the diet (Figure 8C). Mice fed the SUN diet had enriched proportion of n-6 FAs, those fed the LIN and FISH diets are particularly related to n-3 FAs. Mice fed the COC diet preferentially accumulate mono-unsaturated FAs in their livers. FA C18:2 n-6 is accumulated in PPAR*α *deficient mice when compared with wild-type mice. In a same way, the separation between PPAR*α *deficient mice and wild-type mice on PC2 is explained by PPAR*α *target genes (Peci, Cyp4a10, Cyp4a14, Acox1, Acaa1b, Hmgcs2, Cpt2) or genes involved in hepatic detoxification (Cyp3a11). These MFA-based interpretations are in agreement with the results of [31].

The superimposition of prior biological knowledge is presented in Figure 8D. Annotations correlated to PC1 and PC2 are generally linked to energetic metabolism. Most of the GO terms strongly associated with PC1 (i.e, those related to the COC diet) are related to lipid metabolism and particularly 'fatty acid oxidation' (0.7). It is known that hepatic FA catabolism is down-regulated with a saturated diet leading to an accumulation of FA in the liver. This is in accordance with the results discussed by Martin *et al*. [31]. The GO terms associated with PC2 are related to -ose metabolism. They reveal an effect of the diet FA content on glucose metabolism. The associated genes (Ppara, Pklr, Gck, G6pdx, G6pc, Pdk4) also suggest a possible role of PPAR*α *in these mechanisms. These gene expression changes were highlighted in the Table 4 in [31] but not further discussed. It would however be of great interest to precise this putative role of FA diet composition in relation to metabolic syndromes and diabetes.

## Conclusion

Our approach based on MFA in the sense of Escofier-Pagès aims at providing a multivariate data analytic technique for applications in biological systems. It is dedicated to combined 'Omics' data structured into groups and its purpose is also particularly to help on their functional interpretations. MFA is firstly used to simultaneously analyze the structure emerging from the separate analysis of each molecular levels and to supply principal components which summarize parts of the data variability. The common structures are underlined and graphical outputs are provided such that biological meaning becomes retrievable. Partial representations allow the visualization of each 'Omics' point of view. The addition of sample annotations as categorical supplementary variables is used to attach a biological meaning to each component. Functional interpretation is obtained by superimposing biological knowledge on the experimentally interpreted plots. Such work is done by building gene modules from formalized annotations. Modules assembled as sets of genes are projected as supplementary information onto the plane spanned by the two principal components issued from MFA. In this way, we provide a measurement of the correlation between each module and each component. It thus becomes possible to attach functionally meaningful characteristics to each experimentally interpreted component.

With our method, investigation of microarray data is supported by a step-by-step sequence of graphical representations. MFA plots provide a clear visualization of the data. Each plot corresponds to one interpretable entity: Individual Factor Map, Variables Correlation Circle and Groups Representation; all of them sharing closely related dimensions. Exploration and functional interpretation are thus based on the understanding of these dimensions. Moreover, in our approach, annotations are not transformed but are used to create GO modules. GO modules are assembled with the expression values of all its constituting genes. When projecting the corresponding annotation, this allows the consideration of the transcriptome structure of a GO module instead of only appreciate GO annotations via boolean vectors.

We have illustrated our approach on a complex setting which is the study of human brain cancers. Firstly, we focused on a study combining the genome and the transcriptome of gliomas and secondly, an other study related only to the transcriptome of these tumors. The latter one allows us to show that our approach could be applied to a single group of data (classical microarrays), which is the most frequently methodology used in the high-througthput area of biology. The addition of annotations as supplementary gene modules give very good insights into the molecular bases of malignant primary brain tumors. Relevant mechanisms involved in cancer were identified, and more precisely some well defined in glioblastoma (such as alteration of the cell cycle, proliferative behavior and development/cell differentiation).

Using the nutrimouse study, we have tried to show the general applicability of MFA to any investigations needing a comprehensive view of the data. Futhermore, one major advantage of this method is not to be bound to any specific experimental design nor to any type of annotation. Gene modules can therefore be created with any gene related knowledge: processes (GO terms from the three hierarchies, SwissProt keywords), pathways (KEGG, BioCarta, Reactome), structural or promoter informations (TRANSFAC, TRRD and COMPEL [32]).

## Methods

### Brain cancer data sets

Three microarray experiments on human glial tumors were used. We retrieved the corresponding publicly available data sets as 'Series Matrix Files' (GSE1991, GSE2223 and GSE4412) from the Gene Expression Omnibus (GEO) database [33]. Bredel *et al*. studies, noted *Multiple data sets*, were performed respectively at the transcriptome [19] and at the genome level [20] on the same tumor samples (GSE1991 and GSE2223). The one of Freije *et al*., noted *Single data set*, was performed at the transcriptome level only [34] (GSE4412). It is used to illustrate the application of our methodology in a single data framework. We considered the four main types of glial tumors defined by the standard World Health Organization (WHO) classification: O, oligodendrogliomas; A, astrocytomas; OA, mixed oligo-astrocytomas and GBM, glioblastomas. We retrieved, for each data set, only the corresponding hybridization. From an histological point of view, A and GBM are astrocytic tumors, O are oligodendrocytic tumors and OA are mixed tumors. According to WHO grading: GBM are grade IV and O, OA and A are lower grade gliomas.

#### Multi-way glioma data set

We retrieved the CGH-array and transcriptome data corresponding respectively to the genetic alterations and the transcriptomic changes highlighted by Bredel *et al*. (see [35] and [36] supplementary data) for 43 tumor samples (5 A, 8 O, 6 OA, 24 GBM). Measurements were expressed as ratios of the two channels intensities: tumor DNA over sex-matching reference DNA for the CGH-array data and tumor RNA over non-neoplastic brain RNA for the transcriptome data. Classical standardization was performed on the data: ratios of the two channels intensities were *log*_2 _tranformed and mean centered per array. Two matrices of numerical variables were built: *K*_1 _contains the data for the expression study (eX), and *K*_2 _those from the genome investigation (CGH). Each matrix has 43 samples (5A, 8O, 6OA, 24GBM); *K*_1 _has 489 genes and *K*_2 _has 113 genes.

#### Single glioma data set

Data files (Affymetrix HG-U133A and HG-U133B) corresponding to 85 tumor samples (8 A, 11 O, 7 OA, 59 GBM) were selected. Data were prepared as described by [34]. Briefly, intensities were *log*_10 _tranformed and median-centered per array. Probe sets were then filtered to select for a 0.2 coefficient of variation with at least 10% of the samples having an expression intensity > 500. One way analysis of variance (ANOVA) was carried out using the subsequent probe sets and the WHO classification factor corresponding to each tumor type; significance was set equal to 10^-5^. This yielded 615 probe sets, which contain most of the variation in gene expression information across all of the samples. The corresponding data matrix, *K*_3_, has 85 samples and 615 genes.

### Nutrimouse data set

This data set corresponds to a nutrition study in mouse. A full description of the experimental settings is provided in [31] and data are available in the CCA R package [37]. Experimental design has two factors: Genotype and Diet. Genotype includes wild-type (WT) and PPAR*α *knock-out mice (20 and 20). Diet includes 5 levels depending on their fatty acid (FA) composition: a reference diet (REF), a saturated FA diet (COC), an *ω*6 FA-rich diet (SUN), an *ω*3 FA-rich diet (LIN) and a corn/colza/enriched fish oils diet (FISH). Data is composed of two sets of variables: *K*_1 _contains the data for the transcriptome study, and *K*_2 _those for hepatic FA measurements by gas chromatography. Each matrix has 40 samples (mice) (Genotype, 2 levels and Diet, 5 levels); *K*_1 _has 115 genes and *K*_2 _has 21 FA.

### GO annotations

We converted the array probes ID to suitable identifiers for all the data sets. Gene symbol were extracted from the HUGO Gene Nomenclature (HGNC) database [38] using the array probes description provided by GEO and manual searches. The functional annotations of the corresponding genes were retrieved from the Human subset of the Gene Ontology Annotation (GOA) database [39]. We restricted the annotations to focus on the GO biological process (BP) terms only. We used the *true path rule *[40] to associate each gene with all the GO terms subsumed by its annotated terms. The GO BP terms annotating only one gene were not taken into account. The enrichment of each GO term was computed using the NIH-DAVID software . Fisher Exact statistics were calculated based on the whole genome (human or mouse) with corresponding DAVID gene ID as the reference. GO terms were considered enriched for a modifided Fisher Exact *p*-value (EASE score) < 0.05. We assigned a BP to a set of genes, and built modules by compiling the data profiles of the genes involved in the same biological process. To formalize, in the case of two data tables, let *K *denotes the concatenation of the matrices *K*_1 _and *K*_2 _(or only *K*_3 _in the case of single data set framework), and let $K_{BP_{i}}$ denotes the restriction of *K *to the genes associated with the *i*^*th *^BP. The $K_{BP_{i}}$ have several levels: some are very large when others are nested and well-focused. For the *Multiple data sets*, the 288 BPs (corresponding to eX) were made from 2 to 324 genes (the mean number of genes in a biological process is equal to 20.84 and the median is 7) and 82 BPs (corresponding to CGH) were made from 2 to 66 genes (the mean number of genes in a biological process is equal to 9.33 and the median is 4). For the *Single data set*, 1081 BPs were made from 2 to 405 genes (the mean number of genes in a biological process is equal to 14.5 and the median is 4). Data are then divided into four parts: samples description (WHO), data from expression microarrays (eX), data from CGH arrays (CGH) (or only eX in the case of the single data set) and gene annotations (BP).

### Data analysis

Here, we briefly describe MFA and focus on one of its main features: the possibility to add supplementary groups of variables. A schematic of our approach is provided Figure 1. For more information about the mathematical details of MFA, one could read [41].

#### Application of MFA to 'Omics' data tables

We consider the merged data set: *K *= [*K*_1_, *K*_2_,..., *K*_*J*_], where each *K*_*j *_corresponds to an 'Omics' data table. Firstly, separate analysis are performed by principal components analysis (PCA) on each group *j *of variables. Secondly, a global analysis is carried out: each variable belonging to a group *j *is weighted by 1/$\lambda_{1}^{i}$, where $\lambda_{1}^{i}$ denotes the first eigenvalue of the matrix of variance-covariance associated with each data table *K*_*j*_. The rationale of the scaling is that information that is common to the data tables emerges. Besides no data table can, by itself, generate the first dimension of the global analysis. The first dimension's variance of each data table is then equal to one. In such way, MFA provides a balanced representation of each individual according to the joint data table *K*, but also a partial representation of each individual according to each of the group *j *of variables. The corresponding graphical displays (Individual Factor Map and Variables Representation) are read as for PCA. The partial individual *i*^*j *^is on the side of the variables of the group *j *for which it takes high values, and on the opposite side of the variables of the group *j *for which it takes low values. Partial representations of a same individual are all the more close that they do express the same information. And, the balanced representation of an individual *i *is located in the exact barycenter of the points {*i*^*j*^, *j *= 1,..., *J*}. Each category is represented by the center of gravity of the cloud of all its constituting individuals. The representation of the variables is used to describe the dimensions as in PCA. MFA provides also a representation of each matrix of variables (Groups Representation) that allows the visualization of specific and common structures. Consequently, it is possible to get an overall picture of the common structure emerging from the *K*_*j*_.

#### Integration of biological knowledge: adding supplementary groups of variables

By integrating biological knowledge, we want to identify the biological processes that best reflect the molecular changes characterizing the conditions under study. From a biological point of view, a biological process can be seen as a module of genes (set of genes with related molecular data); from a statistical point of view, a biological process can be seen as a group of variables. We formalized the BP modules as $K_{BP_{i}}$ matrices containing the restriction of the whole data set to the genes associated with the BP *i*. Each $K_{BP_{i}}$ denotes a matrix of dimension *n *× *p*_*i*_, where *n *is the number of tumors and *p*_*i *_is the number of genes associated with the BP *i*. We then used one feature of MFA that consists in the addition supplementary groups of variables. These supplementary groups of variables do not participate to the construction of the dimensions. This is essential since a gene belonging to several biological processes would have more importance in the analysis if the groups were active. This feature lies as MFA could be seen as a particular generalized canonical analysis where the general variables are related to the sets of variables as strongly as possible in the sense of the *L*_*g *_measure (instead of the multiple correlation coefficient *R*^2^). The *L*_*g *_measure between one numerical variable *z *and a set of variables $K_{j}$ = {*v*_*k*_, *k *= 1,..., *K*_*j*_} is defined by the inertia of all variables *v*_*k *_projected upon *z*. If *L*_*g*_(*z*, *K*_*j*_) = 0, the variable *z *is not correlated to any variable of the set *K*_*j*_. Due to the MFA weighing, 0 ≤ *L*_*g*_(*z*, *K*_*j*_) ≤ 1 and *L*_*g*_(*z*, *K*_*j*_) = 1 when *z *is the first principal component of *K*_*j*_. Let *F*_*s *_be the dimension of rank *s *(coordinates of the individuals) provided by MFA performed on *K*. The projection of the $K_{BP_{i}}$ is made by means of its scalar product matrix between individuals. This matrix denoted *W*_*i *_is a a (*I *× *I*) matrix (*W*_*i *_= $K_{BP_{i}}{K^{\prime }}_{BP_{i}}$) and can be considered as an element of the space ${\mathbb{R}}^{I^{2}}$. This element is projected on the dimensions of ${\mathbb{R}}^{I^{2}}$ induced by the vectors issued from the matrices *F*_*s*_${F^{\prime }}_{s}$. Given that the coordinates of a group in this representation is interpreted as a measure of relationship related to *L*_*g*_, the two following properties lie: (i) the coordinates are always comprised between 0 and 1, and (ii) a small distance between two groups along the principal component of rank *s *means that these two groups include the structure expressed by *F*_*s *_each one with the same intensity. This representation of the groups is made available by means of a graphical display of the $K_{BP_{i}}$ as points in a scatter plot. It has to be read as follow: the coordinate of a given group is all the more close to 1 than the variables of this group are highly correlated with the dimension issued from the MFA (either positively or negatively). Hence, two groups are all the more close than the structures they induce on the observations are close.

## Authors' contributions

MDT interpreted and analyzed the data, provided drafting of the article, collected and assembled the data. Statistical methodology were defined by SL, FH and MDT. GO modules were computed by MA and MDT. R package FactoMineR has been developped by FH and SL. The biological interpretation was performed by MDT, MA and JM. SL, FH and JM supervised this study and contributed to continuous discussions. All authors have read and approved the final manuscript.
